# Detection of spermatogonial stem cells in testicular tissue of dogs with chronic asymptomatic orchitis

**DOI:** 10.3389/fvets.2023.1205064

**Published:** 2023-06-15

**Authors:** Larena Reifarth, Hanna Körber, Eva-Maria Packeiser, Sandra Goericke-Pesch

**Affiliations:** Reproductive Unit – Clinic for Small Animals, University of Veterinary Medicine Hannover, Foundation, Hannover, Germany

**Keywords:** DAZL, FOXO1, PGP9.5, C-Kit, chronic asymptomatic orchitis, immune orchitis, Infertility, dog

## Abstract

Chronic asymptomatic idiopathic orchitis (CAO) is an important but neglected cause of acquired infertility due to non-obstructive azoospermia (NOA) in male dogs. The similarity of the pathophysiology in infertile dogs and men supports the dog's suitability as a possible animal model for studying human diseases causing disruption of spermatogenesis and evaluating the role of spermatogonial stem cells (SSCs) as a new therapeutic approach to restore or recover fertility in cases of CAO. To investigate the survival of resilient stem cells, the expression of the protein gene product (PGP9.5), deleted in azoospermia like (DAZL), foxo transcription factor 1 (FOXO1) and tyrosine-kinase receptor (C-Kit) were evaluated in healthy and CAO-affected canine testes. Our data confirmed the presence of all investigated germ cell markers at mRNA and protein levels. In addition, we postulate a specific expression pattern of FOXO1 and C-Kit in undifferentiated and differentiating spermatogonia, respectively, whereas DAZL and PGP9.5 expressions were confirmed in the entire spermatogonial population. Furthermore, this is the first study revealing a significant reduction of PGP9.5, DAZL, and FOXO1 in CAO at protein and/or gene expression level indicating a severe disruption of spermatogenesis. This means that chronic asymptomatic inflammatory changes in CAO testis are accompanied by a significant loss of SSCs. Notwithstanding, our data confirm the survival of putative stem cells with the potential of self-renewal and differentiation and lay the groundwork for further research into stem cell-based therapeutic options to reinitialize spermatogenesis in canine CAO-affected patients.

## 1. Introduction

The most frequent clinical finding in infertile male dogs is azoospermia, the complete absence of spermatozoa in the ejaculate (1–4). More detailed, non-obstructive azoospermia (NOA) is most common in male dogs (3, 5–8), but also in other species, such as men (9–11). The role of immune cells in infertility due to NOA received increased attention across a number of species in recent years. In men, focal lymphocytic infiltrations have been detected in 25–30% of testicular biopsies from infertile patients (12–14). Further insights into molecular mechanisms of immune cell infiltration leading to the breakdown of spermatogenesis have been gained from studies on experimental autoimmune orchitis (EAO) in rodent models (12, 13). Immune-cell infiltration has also been described in infertile male dogs and—due to the lack of clinical signs—has been associated with spontaneous autoimmune orchitis (AIO), in mainly individual case reports (8, 15–19). These reports describe histopathological changes such as seminiferous tubular degeneration, atrophy, and parenchymal collapse along with immune cell infiltration in the testicular tissues of affected, however, otherwise asymptomatic dogs (8, 15–20). So far, only one publication identified concomitant autoimmune thyroiditis in 17 of 22 affected dogs of a closed beagle colony (15).

Recently, we characterized a larger collective of testicular biopsies from asymptomatic, but infertile canine NOA patients and confirmed severe histopathological alterations, such as Sertoli cell-only (SCO) syndrome, tubular shadows, and fibrosis, concomitantly with lymphoplasmacytic inflammation of both testes (8). Immune cells, namely plasma and T cells, invade not only the interstitium but also the seminiferous tubules (21). These immune cells overcome and disrupt the blood testicular barrier (22) as earlier described in men and EAO mice (12) and lead to focal or generalized inflammatory changes as well as to a corresponding arrest of spermatogenesis (8). These results provide empirical evidence for the postulate that chronic asymptomatic, possibly immune-cell mediated orchitis (CAO) is ***the*** cause of acquired infertility due to NOA in the dog. As there is still no certainty regarding the etiology of the disease and its development, CAO is considered to be “spontaneous” or rather idiopathic in the dog. With the onset of infertility, a poor prognosis and irreversible damage to the testicular tissue have to be assumed and previous studies failed to identify treatment options with significant changes in fertility outcome or even recovery or restoration of fertility, not only in dogs (23) but also in humans (24). In the case of infertility in men, assisted reproductive technologies, such as intrauterine insemination, *in vitro* fertilization, or intracytoplasmic sperm injection represent the last resort (25, 26).

Another approach to the prophylaxis and treatment of human male infertility is based on the derivation of spermatogonial stem cells (SSCs) from testicular tissues by ectopic or orthotopic transplantation to (re-) initialize spermatogenesis (27). Despite the controversial discussion about whether “stemness potential” is restricted to a particular cell type or dynamically distributed in all types of spermatogonia (28, 29), SSCs derive from primordial germ cells during embryonic development and support tissue maintenance through balancing self-renewal and differentiation as the basic requirement for lifelong spermatogenesis (30–35). Thus, an increasing number of studies examine the use of stem cells for regenerative and reproductive science in domestic animals, too (36). In addition, since the first successful SSC transplantation (SSCT) in a dog (37), there has been a growing number of publications focusing on the efficiency and improvement of SSCT (38–42). Although studies over the past two decades provided important information on specific male germ cell biomarkers in rodents, data regarding dogs are limited (43–50).

Important biomarkers studied in relation to germ cell renewal and differentiation for maintenance of spermatogenesis are PGP9.5 (protein gene product 9.5, also known as UCHL-1), DAZL (DAZ-like, deleted in azoospermia), FOXO1 (foxo transcription factor 1), and the tyrosine-kinase receptor C-Kit (also known as CD117) and its ligand (stem cell factor, SCF). Ubiquitin-dependent proteolysis has been implicated in the control of mammalian gametogenesis (51, 52), and the deubiquitinating enzyme PGP9.5 plays an important role in apoptosis, meiosis, and mitotic proliferation in germ cells (53). Results from earlier studies demonstrated an exclusive expression of PGP9.5 in spermatogonia across species, including mice (51), boars (53, 54), bulls (55), and dogs (43, 47). Different from this, DAZL expression is described in various germ cell populations in several vertebrates and invertebrates (56–58), including dogs (45, 49), pigs (53, 59), and mice (60), and is crucial for the initiation of translation during gametogenesis. FOXO1 and C-Kit expression, however, is limited to specific germ cells within the spermatogonial population. FOXO1 expression is restricted to undifferentiated spermatogonia with stem cell potential in the adult testis of various species, including the dog (50). It controls maintenance and differentiation through a specific network of transcriptional targets, including the receptor tyrosine kinase Ret (61). C-Kit expression is linked to the differentiation of type A spermatogonia (62) in several species, including dogs (45, 63–65).

However, despite the growing number of publications focusing on specific male germ cell biomarkers for the identification of SSCs in several species, there have been no attempts to evaluate a stem cell-based therapeutic approach for the treatment of CAO in male dogs up to now. Furthermore, data regarding the survival of viable stem cell populations in the respective canine testicular tissues are currently completely missing. Consequently, the purpose of this study was to investigate the expression of the canine biomarkers PGP9.5, DAZL, FOXO1, and C-Kit in CAO-affected testicular tissue. We hypothesized that SSCs survive in the canine CAO testis despite the histopathological alterations and lymphoplasmacytic infiltration associated with the ongoing disease. However, since the pathological conditions disturb the complex paracrine, molecular, and cellular interaction between SSCs and the “SSC niche”, we assumed a reduced number of SSCs in the case of CAO compared with normal, healthy testis obtained from normospermic canine patients. Furthermore, the aim was not only to detect but also to characterize the surviving spermatogonial population and to compare our findings to previous studies on canine germ cell development.

## 2. Material and methods

Permission for all samples was received by the respective authorities. The collection of CAO samples for diagnostic purposes was approved by the Dyreforsøgstilsynet Fødevarestyrelsen and by the animal ethics committee of the University of Veterinary Medicine Hannover, Foundation. Testis samples from control animals were taken from dogs that were presented for routine castration for other than health issues. All owners agreed on further use of samples for research purposes.

### 2.1. Animals and grouping

Twenty-five sexually mature, clinically healthy male dogs were included in this study. Fifteen dogs were assigned to the CAO group (CAO) after confirmation of permanent azoospermia. Dogs had a mean age of 5.5 ± 1.9 years (2.6–9.6 years) and represented the following breeds: Collie: *n* = 3, Cairn Terrier, Coton de Tulear, Beagle, and German Shepherd Dog: *n* = 3, and Iceland sheepdog, Jack Russel Terrier, Labrador Retriever, Miniature Poodle, Welsh Corgi Pembroke, and Cane Corso: *n* = 1 each. Part of the dogs were also included in previous studies (8, 66). The remaining ten dogs with a mean age of 4.2 ± 2.8 years (1 – 8.9 years) served as a healthy control group (CG) with normospermic ejaculates according to the reference values (67, 68). Animals of the control group belonged to six different breeds (*n* = 1 each: Boxer, Havanese, Malteser, Boston Terrier, and Chihuahua; and *n* = 3 Beagle), and two male dogs were mongrels.

### 2.2. Study design, sample collection, and processing

Detailed clinical and histological procedures to identify azoospermia, to verify non-obstructive azoospermia (NOA), to rule out bacterial infectious causes, and to verify immune cell infiltration (CAO) had been described earlier (8, 21). Briefly, clinical and andrological examinations including semen collection and analysis, ultrasound of the genital tract, and determination of the alkaline phosphatase (AlP) in seminal plasma (>5000 U/l) were performed to confirm NOA. Further bacteriological examination of the ejaculate and serological testing for *Brucella canis* was used to rule out infection, and endocrine analysis (testosterone/estradiol-17ß/thyroxin/free thyroxin/cTSH/thyroid antibodies) was used to rule out pretesticular origin of NOA. Scissor biopsies from both testes were obtained for histological assessment, and CAO was confirmed in all included dogs (8). Samples of the CAO group were further differentiated based on the histological appearance, namely the arrest of spermatogenesis: early arrest (*n* = 5) with Sertoli-cell only (SCO) or spermatogenesis arrested on spermatogonia; and late arrest (*n* = 10) with spermatogenesis arrested on spermatocytes or later stages of spermatogenesis. Testicular tissues in CG (*n* = 10) were collected during surgical castration performed upon the owner‘s request due to other reasons than testicular disease.

### 2.3. Preparation of testicular tissues

The preparation of testicular tissues for histology, mRNA, and protein extraction/analysis was as previously described (69). Briefly, the samples were partly placed in RNAlater^®^ (Qiagen GmbH, Hilden, Germany) and frozen at−80°C, while the other part was fixed in Bouin's solution for 24 h, washed several times in 70% ethanol and subsequently embedded in paraffin.

### 2.4. Quantitative real-time PCR

Isolation of total RNA from the RNAlater^®^-immersed testicular tissues of each dog was performed using TRI reagent^®^ (Sigma Aldrich, St. Louis, Missouri, USA) following the manufacturer's instructions. RNA concentration and quality were measured using a NanoPhotometer^®^ NP80 (IMPLEN, Munich, Germany). All RNA samples were treated with Protector RNase Inhibitor (Sigma Aldrich) and stored at-80°C until used. Full-length first strand cDNA-synthesis was performed using 200 ng/μl RNA and the RevertAidFirst Strand cDNA Synthesis kit (#K1622, Thermo Fisher, Waltham, MA, USA), including DNase treatment, according to the manufacturer's protocol. In order to determine the expression of *PGP9.5, DAZL, FOXO1*, and *C-Kit*, primer sets for RT-qPCR were developed using known sequences available from GenBank (Table 1). *Glyceraldehyde-3-phosphate dehydrogenase* (*GAPDH*), *hypoxanthine guanine phosphoribosyltransferase* (*HPRT)*, and β*-actin* were evaluated for use as housekeeping genes for endogenous control. Finally, *GAPDH* and *HPRT* were chosen as reference genes as they showed the slightest variation in mRNA expression.

**Table 1:** Sequences of primers for RT-PCR and RT-qPCR, amplicon length, efficiency, and accession number (for, forward; rev, reverse).

	**Oligonucleotide sequence (5‘-3‘)**	**Amplicon length (bp)**	**Efficiency**	**Accession number**
GAPDH		228	2.05	NM_001003142
for	GGCCAAGAGGGTCATCATCTC			
rev	GGGGCCGTCCACGGTCTTCT			
HPRT		94	2.05	NM_001003357.2
for	TGACACTGGGAAAACAATGCA			
rev	GGTCCTTTTCACCAGCAAGCT			
DAZL		173	2.1	XM_038432216.1 XM_038432215.1 XM_038432214.1
for	ATCACGGATCGAACTGGTGTGT			
rev	AAAGGACGAGGCTGCACATGA			
PGP9.5		118	2.1	XM_038661918.1 XM_038480474.1
for	CCATCGGGCTTATCCATGCAG			
rev	TTTGCTCGGTCTTCAGGGGA			
FOXO1		172	2.06	XM_038434728.1 XM_022410130.2
for	ATGGTCAAGAGCGTGCCCTAC			
rev	CACTCTTGCCTCCCTCTGGAT			
C-Kit		64	2.1	NM_001003181
for	CCAGTGTGTGGTTGCAGGAT			
rev	CTCAGCTCCTGGACAGAAATACC			

Relative mRNA expression (RT-qPCR) was investigated as described earlier (66, 70): cDNA was added to 8μl of FastStart Essential DNA Green Master (Roche Diagnostics GmbH, Mannheim, Germany), 1μl of the forward and reverse primer (10pmol) (Table 1), and 2μl of sterile Aqua bidest. RT-qPCR was performed with the following cycling conditions for all genes except for *FOXO1* and *HPRT*: 95°C for 10 min, followed by 45 cycles of 95°C for 10 s, 60°C for 10 s, 72°C for 10 s, and melting curve with 80°C. For *FOXO1* and *HPRT*, cycling conditions were slightly modified with the annealing temperature being 57°C. All samples were run in triplicates using a LightCycler^®^96 real-time PCR system (Software version 1.1.0.1320, Roche Diagnostics GmbH, Mannheim, Germany). Calculation of PCR efficiencies of target and reference genes was performed by using a relative standard curve derived from a triplet RT-qPCR run of a 2-fold dilution series (1:2- 1:128) of pooled cDNA samples, whereas the efficiency (E) was E= 10(-1/m) with “m” being the slope of the linear regression line. For the evaluation of the RT-qPCR results, the efficiency-corrected relative quantification according to Pfaffl (2001) (71) was modified and extended taking both reference genes into account as described previously (72). The specificity of the primers for *PGP9.5, DAZL, FOXO1*, and *C-Kit* used in RT-qPCR was checked by using BLAST (https://blast.ncbi.nlm.nih.gov/Blast.cgi), and the results were confirmed by sequencing of PCR products (Microsynth AG, Balgach, Switzerland).

### 2.5. Immunohistochemistry and evaluation of PGP9.5, DAZL, FOXO1, and C-Kit staining

The paraffin-embedded tissue was cut in serial sections of 2 μm thickness and placed on microscopic slides. Preparation and pretreatment of the tissue were performed according to the protocol in earlier studies (73, 74). After deparaffinization and rehydration in xylene and ethanol, the antigen was retrieved by boiling it in 0.01 M sodium citrate buffer at pH 6. After the sections were rinsed in distilled water, the tissue was treated with 3% H_2_O_2_ to block endogenous peroxidase activity. For PGP9.5, non-specific protein binding was blocked with 3% bovine serum albumin (BSA, VWR Life Science, Solon, OH, USA) and 10% horse serum (S-2000, RRID: AB_2336617, Vector Laboratories), and slides were subsequently incubated with the PGP9.5 monoclonal mouse anti-human antibody (7863-1004, RRID: AB_620256, AbD Serotec, Raleigh, NC, USA, dilution 1 x 10^−3^ μg/μl) overnight at 4°C. ICC buffer only was used as negative control and an irrelevant mouse IgG (I-2000, RRID: AB_2336354, Mouse IgG, Control Antibody, Vector Laboratories) as isotype control. For FOXO1, 10% goat serum in ICC buffer was used for blocking followed by incubation with the FOXO1 monoclonal rabbit anti-human antibody (C29H4, #2880, RRID: AB_2106495, CST, Danvers, MA, USA, dilution 0, 44 x 10^−3^ μg/μl) overnight at 4°C. ICC buffer only served as negative control and an irrelevant rabbit IgG (I-1000, RRID: AB_2336355, Rabbit IgG, Control Antibody, Vector Laboratories) as isotype control. Following several washing steps, the respectively treated slides were incubated with SuperVision-2 HRP Enhancer (DCS, Innovative Diagnostik Systeme, Hamburg, Germany) and HRP Polymer (DCS, Innovative Diagnostik Systeme) for 20 min each. Immunopositive signals were visualized with DAB (DCS Chromoline, Innovative Diagnostik Systeme) according to the manufacturer's instructions. Slides were counterstained with hematoxylin after Mayer, dehydrated in increasing alcohol concentrations, and mounted with Roti^®^ Histokitt II (Roth AG, Arlesheim, Switzerland). IHC against DAZL was performed with slight modifications of the protocol mentioned above. For DAZL, non-specific protein binding was blocked with 10% goat serum (S-1000, RRID: AB_2336615, Vector Laboratories, Burlingame, CA, USA) in ICC buffer (1.2 g Na_2_HPO_4_, 0.2 g KH_2_PO_4_, 0.2 g KCl, 8.0 g NaCl, and 3ml Triton ad 10000 ml), and slides were subsequently incubated with the DAZL polyclonal rabbit anti-mouse antibody (ab34139, RRID:AB_731849, Abcam, Cambridge, UK, dilution 2 x 10^−3^ μg/μl) overnight at 4°C. On the next day after washing three times with ICC buffer, samples were incubated with a goat anti-rabbit secondary antibody (BA-1000, RRID: AB_2313606, Vector Laboratories). Immunoreactivity was visualized by an immunoperoxidase method according to the manufacturer's instruction (VECTASTAIN PK-6100 Rabbit IgG Elite ABC Kit, RRID: AB_2336819, and Vector Nova-RED Substrate Kit SK-4800, RRID: AB_2336845, Vector Laboratories). ICC buffer only was used as negative control, and an irrelevant rabbit IgG antibody (I-1000, RRID: AB_2336355, Rabbit IgG, Vector Laboratories) in the respective protein concentration was used as isotype control.

For C-Kit, following the abovementioned treatment, the samples were incubated with 10% goat serum (S-1000, Vector Laboratories) in 3% bovine serum albumin to block unspecific antibody binding and then incubated with a polyclonal rabbit anti-human CD117/c-kit antibody (A4502, RRID: AB_2335702, Dako Denmark A/S, Glostrup, Denmark, 0, 208 μg/μl) overnight at 4°C. Following several washing steps, the slides were incubated with SuperVision-2 HRP Enhancer (DCS, Innovative Diagnostik Systeme, Hamburg, Germany) and HRP Polymer (DCS, Innovative Diagnostik Systeme) for 20 min each. Visualization was performed using DAB (DCS Chromoline, Innovative Diagnostik Systeme) according to the manufacturer's instructions. ICC buffer only was used as a negative control and an irrelevant rabbit IgG antibody (I-1000, RRID: AB_2336355, rabbit IgG, Vector Laboratories) in the respective protein concentration was used as isotype control.

All slides for each antibody were performed simultaneously to avoid bias regarding experimental conditions.

For histological evaluation of PGP9.5, DAZL, and FOXO1, immunopositive signals were counted using an Olympus BX41TF Microscope (Olympus^®^, Shinjuku, Japan) with an Olympus DP72 camera (Olympus Corporation, Tokyo, Japan) and the Olympus cellSense Dimension Software (version 2.1, Olympus Corporation, Tokyo, Japan). In CAO samples, 20 randomly chosen fields of vision of each dog, 10 from the right and the left testis biopsy, respectively, were evaluated at 200-fold magnification. Similarly, 20 randomly selected fields of vision of each dog from CG were counted and served as a comparison.

Due to a diffuse and weak expression pattern in C-Kit staining, adjacent immunopositive cells were difficult to distinguish from each other and other cells, especially in cases of CAO (**Figure 12**). Therefore, immunopositive staining was evaluated descriptively using an Olympus BX41TF Microscope (Olympus^®^, Shinjuku, Japan) at 200-fold and 400-fold magnification.

### 2.6. Protein extraction and Western blot analyses

A Western blot analysis was performed to confirm the specificity and reactivity of primary antibodies in canine tissues as described earlier (73, 75). Frozen canine total testicular tissue (approximately 0.8g) obtained from a healthy adult dog with normal spermatogenesis was dissolved in PBS with a protease inhibitor cocktail (Roche^®^ Diagnostics, Basel, Switzerland), pulverized and homogenized using an Ultraturrax (IKA^®^, Staufen, Germany). After boiling for 10 min with 0.15 g of dodecyl sulfate sodium salt, the insoluble material was removed by centrifugation at 1200 x *g* for 10 min at 4°C. The protein concentration of the supernatant was quantified spectrophotometrically using a NanoPhotometer^®^ NP80 (IMPLEN, Munich, Germany). The obtained total protein was aliquoted and stored at −80°C. Tissues of appropriate positive controls were treated appropriately (Table 2). Canine testis protein and corresponding positive control proteins were used following denaturation by heating at 95°C for 10 min in a Laemmli sample buffer in the water bath. Protein separation was achieved by a 4–20% gradient sodium dodecyl sulfate-polyacrylamide gel (Mini-Protean^®^ TGX^TM^ Gels, Bio-Rad Laboratories, Hercules, California, USA) electrophoresis. After blotting the proteins on a nitrocellulose membrane (Trans-Blot^®^ Turbo^TM^ Transfer Pack, Bio-Rad Laboratories) using a blotting system (Trans-Blot^®^ Turbo ^TM^ Transfer System, Bio-Rad Laboratories), unspecific binding sites were blocked by incubating the membrane for 1 h at room temperature in 5% skimmed milk powder in TBS (Tris-buffered saline). Following several washing steps with TBST (TBS with 0.05% Tween), the membrane was incubated with the primary antibody in 5% skimmed milk powder in TBS for 2 h. After being washed in TBST, the membrane was incubated at room temperature for 1 h with the secondary antibody (biotinylated goat anti-rabbit IgG antibody BA-1000/RRID:AB_2313606 or horse anti-mouse IgG antibody BA-2000/RRID:AB_2313581, Vector Laboratories). Signals were visualized using Clarity^TM^ Western ECL Blotting Substrate (Bio-Rad Laboratories) according to the manufacturer's instructions and a ChemiDoc™ Imaging Systems with Image Lab™ Touch Software (Image Lab 6.0.1, Bio-Rad Laboratories). A mouse IgG nonsense antibody (I-2000, RRID: AB_2336354, Control Antibody, Vector Laboratories) or rabbit IgG nonsense antibody (I-1000, RRID: AB_2336355, Control Antibody, Vector Laboratories) in the same concentration as the primary antibody served as isotype control. Table 2 provides an overview of the use of primary antibodies, secondary antibodies, and positive controls.

**Table 2:** Overview of reagents and dilutions used in the Western blot; SKU, Stock Keeping Unit; IC, Isotype control; ^*^Abcam, Cambridge, UK; ^**^Bio-Rad Laboratories, Hercules, California, USA; ^***^Agilent Technologies, Santa Clara, California, USA; ^****^Cell Signaling Technology, Danvers, Massachusetts, USA.

**Antibody**	**RRID: AB_ Cat#:**	**Source**	**Clonality**	**Concentration (μg/μl)**	**Positive control**	**IC**	**Secondary Antibody**	**Dilution**
DAZL	731849 ab34139^*^	Rabbit	Polyclonal	1 x 10^−2^	Murine testicular tissue	Rabbit IgG	Goat anti-rabbit (BA-1000)	1:500
PGP9.5	620256 7863-1004^**^	Mouse	Monoclonal	2 x 10^−3^	Rat brain tissue	Mouse IgG	Horse anti-mouse (BA-2000)	1:1000
C-Kit	2335702 4502^***^	Rabbit	Polyclonal	5.2 x 10^−2^	Canine mast cell tumor	Rabbit IgG	Goat anti-rabbit (BA-1000)	1:1000
FOXO1	2106495 #2880^****^	Rabbit	Monoclonal	2.9 x 10^−4^	Murine testicular tissue	Rabbit IgG	Goat anti-rabbit (BA-1000)	1:500

### 2.7. Statistical analysis

GraphPad Prism7 software (GraphPad Software, Inc., La Jolla, CA, USA) and Microsoft Excel (Version 16.65, Microsoft, Redmond, WA, USA) were applied for statistical analysis. Values at a level of *p*≤0.05 were considered to be statistically significant. The aim of our study was to identify significant differences between CAO and CG regarding their PGP9.5, DAZL, FOXO1, and C-Kit mRNA and protein expressions.

As the Shapiro–Wilk test did not confirm normal distribution for the mRNA expression (ratio) of *PGP9.5, DAZL*, and *C-Kit*, the Mann–Whitney test was used to test for significant differences between CAO and CG. For *FOXO1*, normal distribution was verified and therefore an unpaired *t*-test was applied. CAO samples were furthermore differentiated into early (*n* = 5) and late arrest of spermatogenesis (*n* = 9), and the results were compared with CG. Regarding *DAZL* group comparison (CAO vs. CG), log-transformed data revealed normal distribution. Subsequently, an ANOVA followed by Tukey's multiple comparisons test using log-transformed data was used to test for significant differences between early and late arrest as well as CG. As the Shapiro–Wilk test did not confirm normal distribution for *PGP9.5, C-Kit*, and *FOXO1*, a non-parametric ANOVA (Kruskal–Wallis test) was applied for overall group comparison (early CAO vs. late CAO vs. CG) followed by Dunn's multiple comparison test in case of the Kruskal–Wallis test revealing a *p*-value of ≤0.05. For comparative reasons, all mRNA expressions (ratios) are presented as geometric mean and dispersion factor [xg (DF)].

For histological evaluation of protein expression of PGP9.5, DAZL, and FOXO1, immunopositive signals in 20 randomly chosen fields of vision (F) were counted and compared. Again, CAO samples were further differentiated into early (*n* = 5) and late arrest of spermatogenesis (*n* = 7), and the results were compared with CG. In all three groups, immunopositive results of fields of vision (F) were evaluated independently and collected in two numerical data sets (CAO vs. CG; CAO early vs. CAO late vs. CG). In addition, the expression patterns of C-Kit during normal and disturbed spermatogenesis were evaluated and compared descriptively.

In the first step, the Shapiro–Wilk test was applied to test for normal distribution of the respective PGP9.5, DAZL, and FOXO1 protein expression obtained from the left and right testes (CAO). After normal distribution was verified, the paired *t*-test was applied for PGP9.5, FOXO1, and DAZL (F) data to prove whether the respective data differed depending on the localization (right/left testis CAO). As no significant differences were identified, the results obtained from both the testes of each dog and the parameter were summarized. The obtained results in CAO samples were compared with the results obtained in CG samples. As the applied Shapiro–Wilk test confirmed normal distribution, data are presented as arithmetic mean and standard deviation ($\bar{x}\pm$SD). An unpaired *t*-test was used to test for significant differences in PGP9.5, DAZL, and FOXO1 protein expression between CAO and CG.

When differentiating CAO into early and late arrest and comparing results to CG, the Shapiro–Wilk test revealed normal distribution for log-transformed data of PGP9.5 and DAZL protein expression, and an ANOVA followed by Tukey's multiple comparisons test was used for overall group comparison. For FOXO1, the Shapiro–Wilk test did not verify normal distribution, and consequently, non-parametric ANOVA (Kruskal–Wallis test) was applied to test for significant differences between the groups (early CAO vs. late CAO vs. CG) followed by Dunn's multiple comparison test in case of the Kruskal–Wallis test revealing a *p*-value of ≤0.05. Again, for comparative reasons, all results of group comparison are presented as geometric mean and dispersion factor [${\bar{x}}_{\text{g}}$ (DF)].

## 3. Results

### 3.1. PGP9.5 mRNA and protein expression

**Figure 1:**
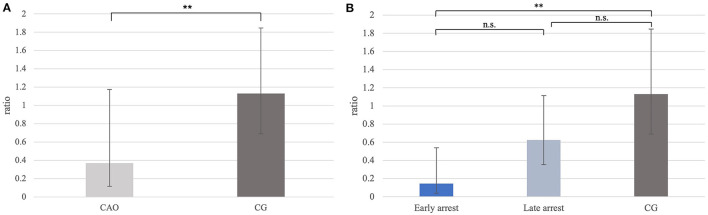
**(A)** *PGP9.5* mRNA expression (ratio) in groups of dogs suffering from immune-mediated orchitis (CAO) and healthy control dogs (CG). The results are presented as geometric mean and dispersion factor [$\bar{x}$g (DF)]. Datasets with asterisks differ significantly: ^**^p = 0.0012. **(B)** *PGP9.5* mRNA expression (ratio) in groups of early and late arrest (CAO) and control dogs (CG). The results are presented as geometric mean and dispersion factor [$\bar{x}$g (DF)]. Datasets with asterisks differ significantly: ^**^p = 0.0028.

Ratios for *PGP9.5* mRNA expression differed significantly between CAO and CG with a significantly lower *PGP9.5* expression in CAO compared with CG (*p* = 0.0012; Figure 1A). Furthermore, the statistical comparison between mRNA expressions in early arrest, late arrest, and CG revealed a significant difference (*p* = 0.0012) with early arrest showing significantly lower *PGP9.5* expression compared with CG (*p* = 0.0028, Figure 1B).

**Figure 2:**
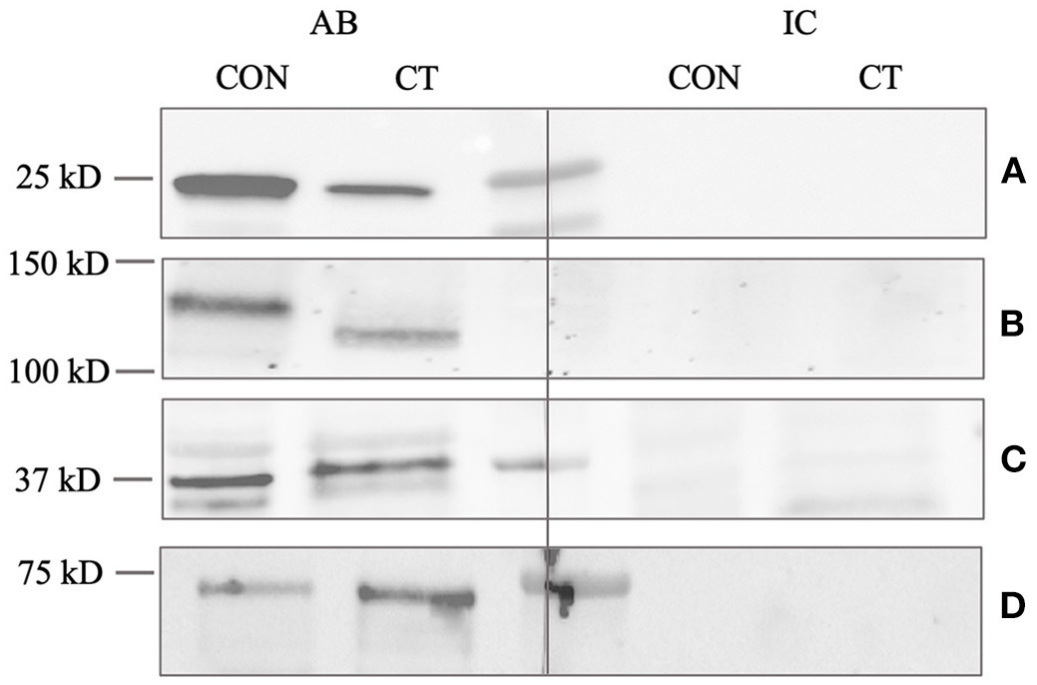
Western blot analysis of **(A)** PGP9.5, **(B)** C-Kit, **(C)** DAZL, and **(D)** FOXO1. CT (canine testis) indicates adult, healthy canine testis tissue. CON (control) indicates an appropriate positive control, either proposed by the manufacturer or demonstrated by previous studies (45, 76). AB indicates the antibody and IC the isotype control. Protein size in kilodalton (kD) is given on the left.

To confirm the reactivity of the PGP9.5 antibody, a Western blot was performed using testicular tissue and rat brain tissue as positive controls. The molecular weight of the canine band was equally located to the positive control at 25 kD (Figure 2A). No specific immunoreactive band was visible in the negative and isotype controls from the canine testes and positive control.

**Figure 3:**
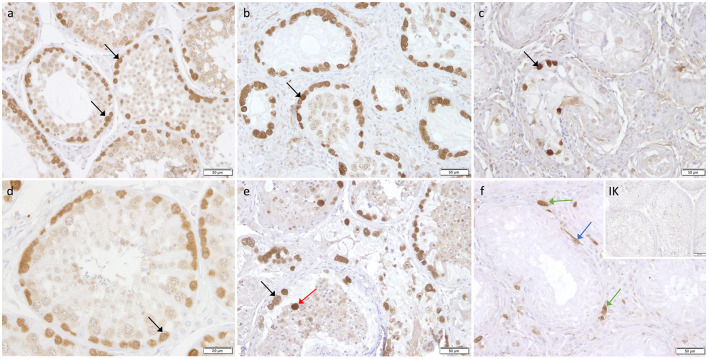
Immunostaining against PGP9.5 **(a–f)** in CG and CAO dogs (200x magnification, **(d)** 400x magnification). **(a, d)** CG, **(b, e)** late arrest, **(c, f)** early arrest. Spermatogonia (*black arrow*); Leydig cell (*green arrow*); peritubular myoid cell (*blue arrow)*; detached spermatogonia (*red arrow*).

**Figure 4:**
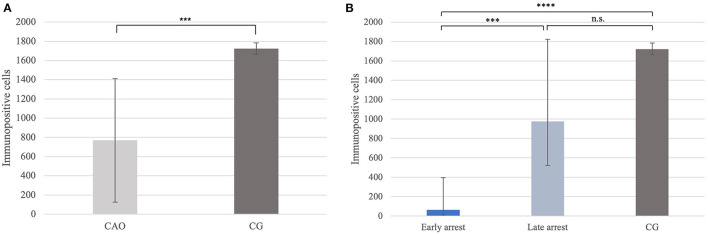
**(A)** PGP9.5 protein expression evaluated as the number of immunopositive cells in 20 fields of vision in groups of dogs suffering from immune-mediated orchitis (CAO) and healthy control dogs (CG). The results are presented as arithmetic mean and standard deviation ($\bar{x}\pm$SD). Datasets with asterisks differ significantly: ^***^p = 0.0002. **(B)** PGP9.5 protein expression evaluated as the number of immunopositive cells in 20 fields of vision in groups of early and late arrest (CAO) and control dogs (CG). The results are presented as geometric mean and dispersion factor [$\bar{x}$g (DF)]. Datasets with asterisks differ significantly: ^****^p = 0.0001; ^***^p = 0.0005.

Immunohistochemistry revealed specific immunopositive staining located in the nuclei and cytoplasm of spermatogonia and early stages of spermatocytes located at the basal membrane of the seminiferous tubules in both groups (Figure 3). However, PGP9.5^+^ staining was restricted to germ cells attached to the basal membrane within the seminiferous tubules of CG, and some immunopositive cells in CAO were detached (Figure 3E). Additionally, some peritubular cells and Leydig cells in the interstitial compartment were stained with PGP9.5^+^ in CAO, especially around the tubules with early arrested spermatogenesis (Figure 3F), but not in CG. The total number of PGP9.5^+^ cells differed significantly between CAO and CG (*p* = 0.0002), with a significantly lower PGP9.5^+^ expression in CAO samples (Figure 4A). Differences between early and late arrest as well as CG revealed significance (*p* = 0.0002). The subsequent Dunn's test identified significant differences between samples with early arrest compared with late arrest (*p* = 0.0005) and with CG (*p* < 0.0001), with fewer PGP9.5^+^ signals in the early arrest group. No significant difference was observed between late arrest and CG (Figure 4B).

### 3.2. DAZL mRNA and protein expression

**Figure 5:**
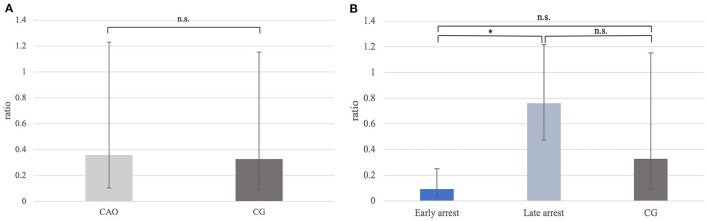
**(A)** *DAZL* mRNA expression (ratio) in groups of dogs with immune-mediated orchitis (CAO) and healthy control dogs (CG). The results are presented as geometric mean and dispersion factor [$\bar{x}$g (DF)]. **(B)** *DAZL* mRNA expression (ratio) in dogs with CAO divided into early and late arrest and control dogs (CG). The results are presented as geometric mean and dispersion factor [$\bar{x}$g (DF)]. Datasets with asterisks differ significantly: ^*^p = 0.0212.

Ratios (mRNA expression) for *DAZL* did not differ significantly between CAO and CG (Figure 5A). However, comparing the groups of early and late arrest to CG, a significant overall difference was found (*p* = 0.0060) with lower *DAZL* expression in early arrest samples compared with late arrest (*p* = 0.0212), but no differences were found between early or late arrest and CG (Figure 5B).

Specificity and reactivity of the DAZL antibody were proven using murine testicular protein as a positive control (Figure 2C). The molecular weight of the specific canine band was equally located to the murine control at 37 kD.

**Figure 6:**
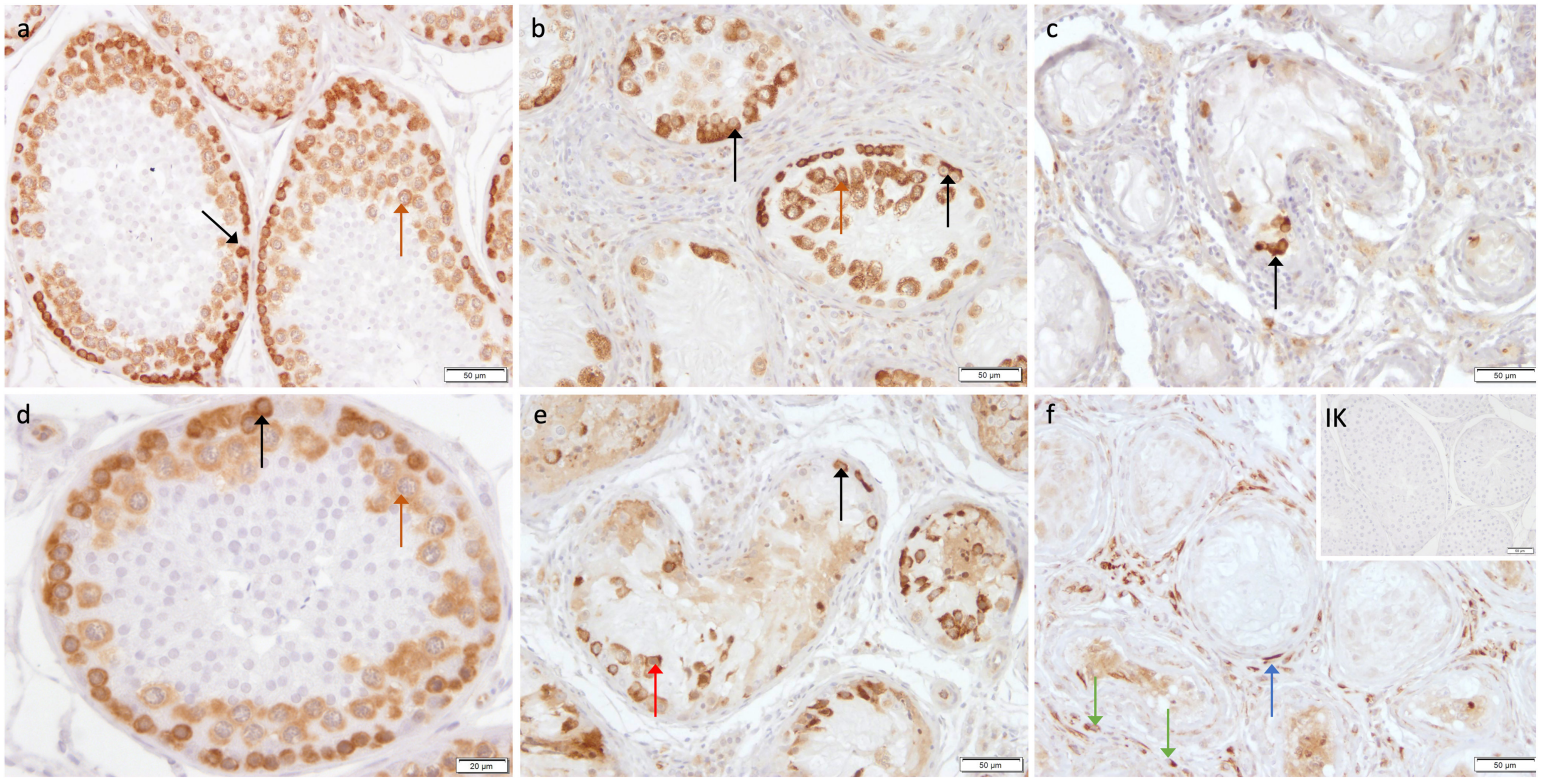
Immunostaining against DAZL **(a–f)** in CG and CAO dogs (200x magnification, **(d)** 400x magnification). **(a, d)** CG, **(b, e)** late arrest, **(c, f)** early arrest. early arrest. Spermatogonia (*black arrow*); spermatocyte (*orange arrow);* Leydig cell (*green arrow*); peritubular myoid cell (*blue arrow)*; detached spermatogonia (*red arrow*).

The cytoplasm of spermatogonia in CAO and CG samples, as well as the cytoplasm of primary spermatocytes, was stained immunopositive against DAZL (Figure 6). In the interstitial compartment, DAZL^+^ staining was identified predominantly in blood vessels and occasionally in the cytoplasm of Leydig cells in both groups. In addition, single DAZL^+^ signals were detected in the peritubular myoid cells in CAO, with individual differences in staining intensity between the samples (Figure 6F).

**Figure 7:**
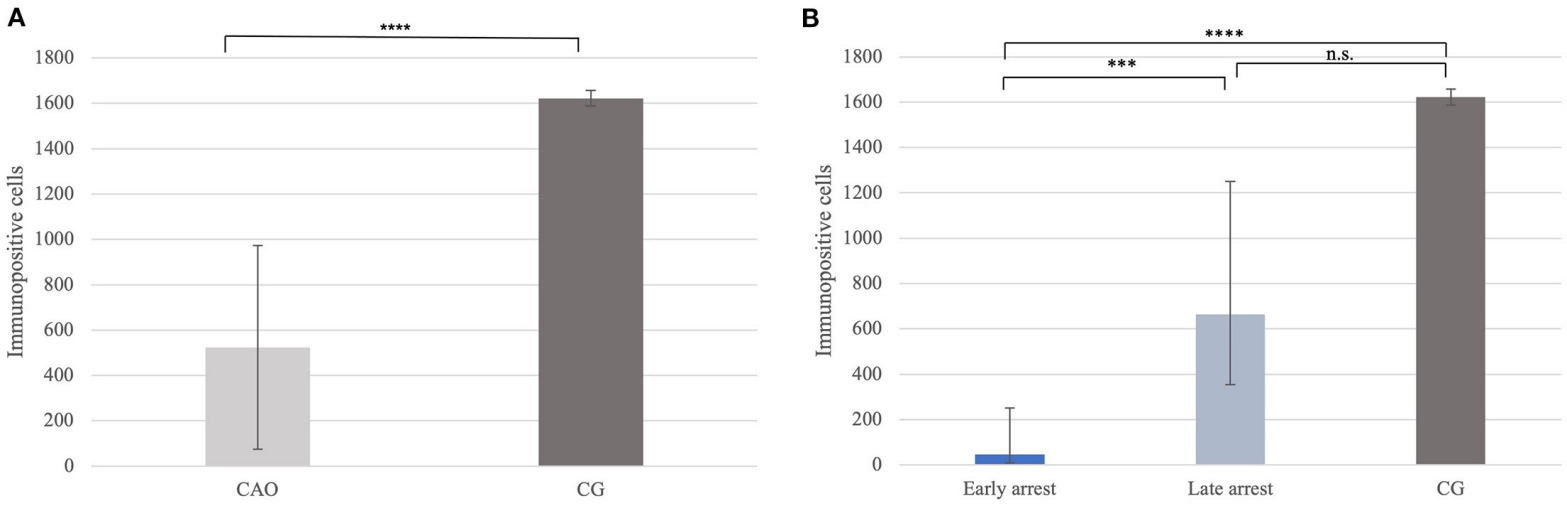
**(A)** DAZL protein expression evaluated as the number of immunopositive cells in 20 fields of vision in groups of dogs suffering from immune-mediated orchitis (CAO) and healthy control dogs (CG). The results are presented as arithmetic mean and standard deviation ($\bar{x}\pm$SD). Datasets with asterisks differ significantly: ^****^p < 0.0001. **(B)** DAZL protein expression evaluated as the number of immunopositive cells in 20 fields of vision in groups of early and late arrest (CAO) and control dogs (CG). The results are presented as geometric mean and dispersion factor [$\bar{x}$g (DF)]. Datasets with asterisks differ significantly: ^****^p < 0.0001; ^***^p = 0.0004.

Significantly fewer DAZL^+^ signals were counted in CAO compared with CG (*p* < 0.0001; Figure 7A). Furthermore, differences between early and late arrest and CG were significantly different (*p* < 0.0001), with the results for early arrest differing significantly different from CG (*p* < 0.0001) and late arrest (*p* = 0.0004). No significant difference was observed between late arrest and CG (Figure 7B).

### 3.3. FOXO1 mRNA and protein expression

**Figure 8:**
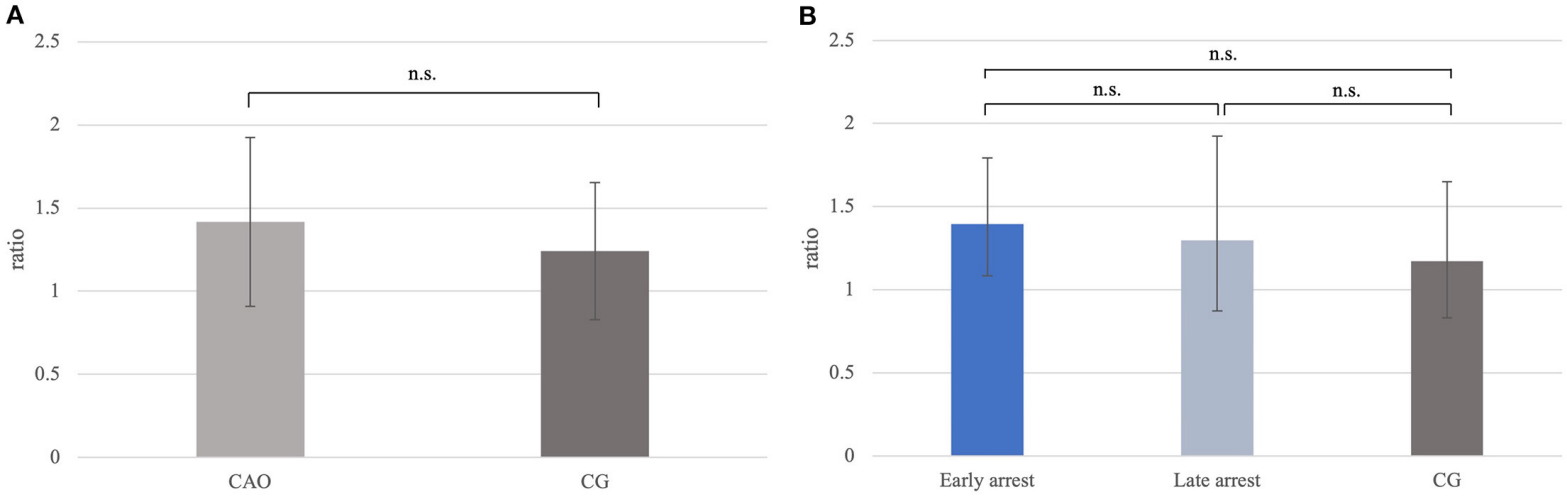
**(A)** *FOXO1* mRNA expression (ratio) in groups of dogs with immune-mediated orchitis (CAO) and healthy control dogs (CG). The results are presented as geometric mean and dispersion factor [$\bar{x}$g (DF)]. **(B)** *FOXO1* mRNA expression (ratio) in dogs with CAO divided into early and late arrest and control dogs (CG). The results are presented as geometric mean and dispersion factor [$\bar{x}$g (DF)].

Ratios for *FOXO1* mRNA expression did not differ significantly between CAO and CG (Figure 8A), nor generally between early and late arrest as well as CG (Figure 8B).

The specificity and reactivity of the FOXO1 antibody were demonstrated by the Western blot using the testicular tissue and an appropriate positive control (Figure 2D). The canine band was located at the same molecular weight (72–75 kD) as the positive control.

**Figure 9:**
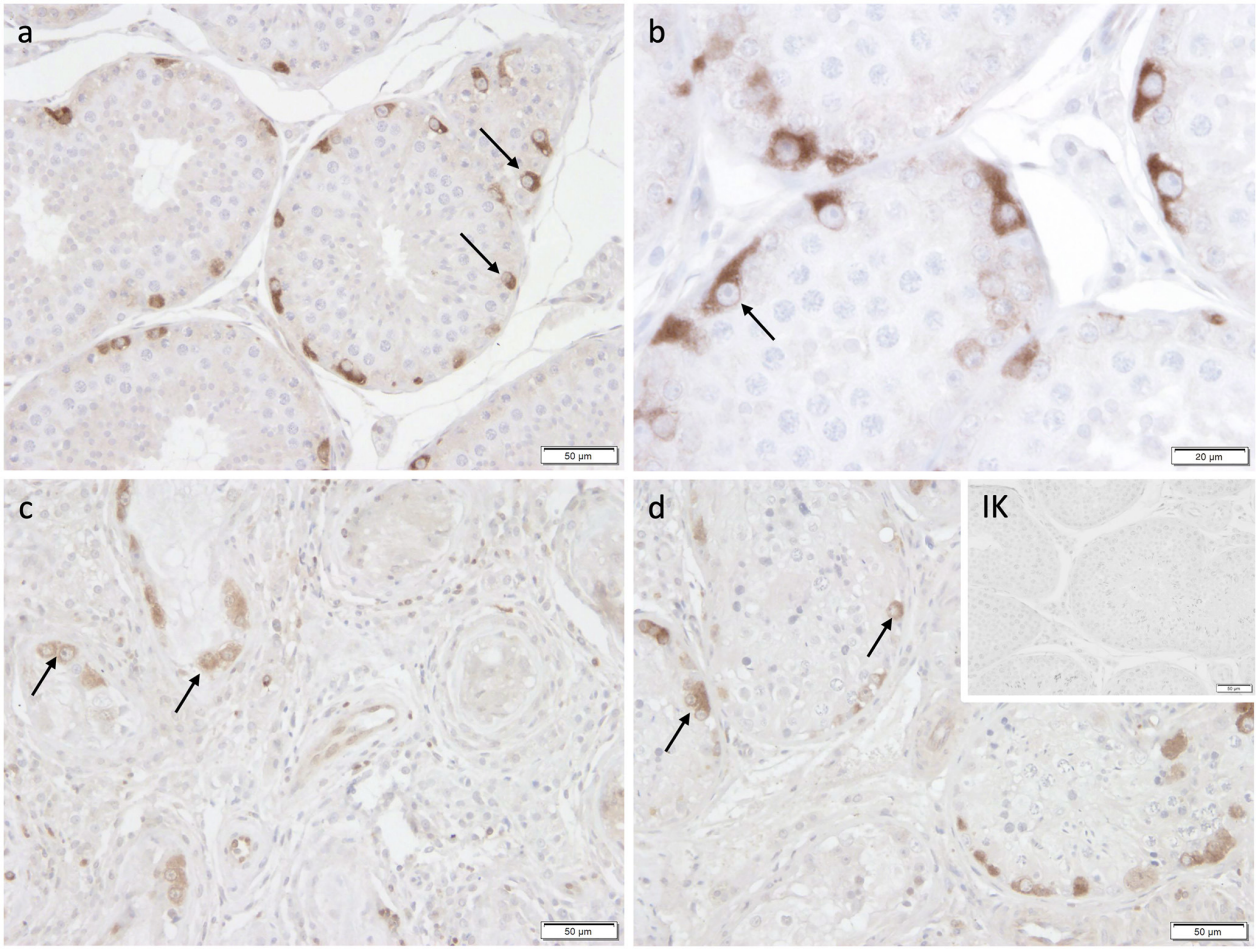
Immunostaining against FOXO1 **(a-d)** in CG and CAO dogs (200x magnification, **(d)** 400x magnification). **(a, b)** CG, **(c, d)** CAO [**(c)** early arrest; **(d)** late arrest]. Spermatogonia (*black arrow*).

Immunopositive staining against FOXO1 was specifically found in the cytoplasm of spermatogonia in CAO and CG (Figure 9) with the staining intensity differing considerably between the samples, but independent of the group (CAO/CG). FOXO1^+^ expression was limited to a specific subgroup within the spermatogonial population with FOXO1^+^ spermatogonia characterized by a prominent round nucleus and extensive cytoplasm (Figure 9).

**Figure 10:**
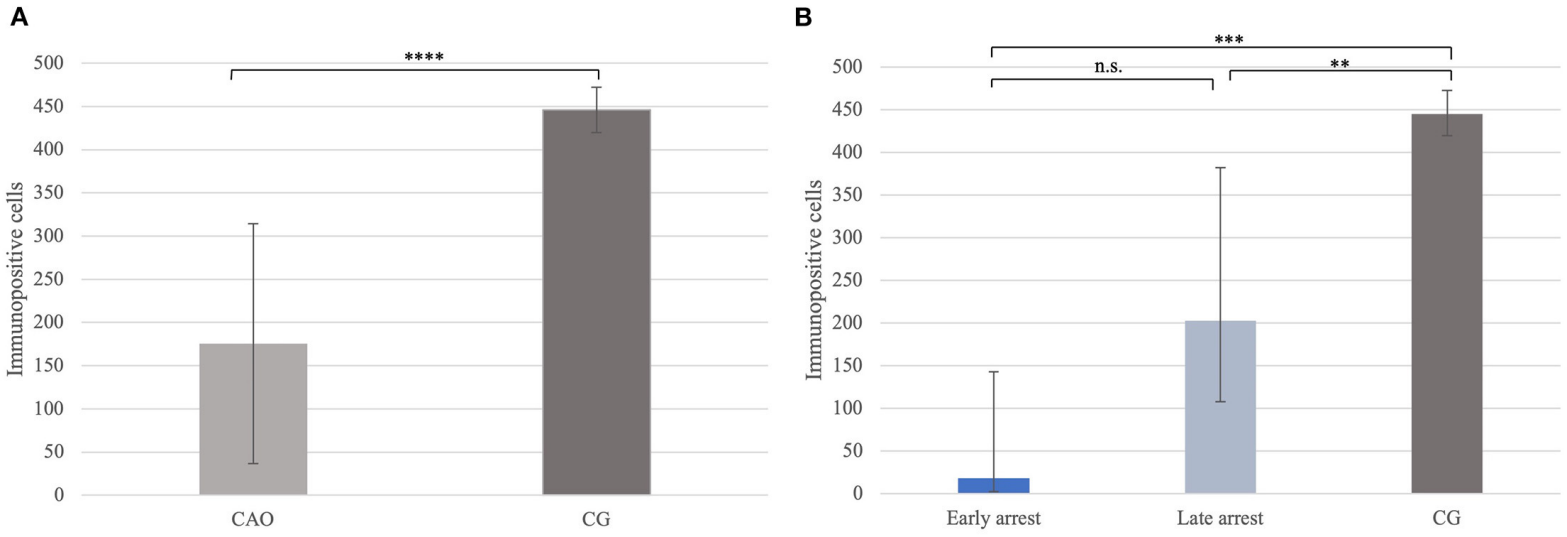
**(A)** FOXO1 protein expression evaluated as the number of immunopositive cells in 20 fields of vision in groups of dogs suffering from immune-mediated orchitis (CAO) and healthy control dogs (CG). The results are presented as arithmetic mean and standard deviation ($\bar{x}\pm$SD). Datasets with asterisks differ significantly: ^****^p < 0.0001. **(B)** FOXO1 protein expression evaluated as the number of immunopositive cells in 20 fields of vision in groups of early and late arrest (CAO) and control dogs (CG). The results are presented as geometric mean and dispersion factor [$\bar{x}$g (DF)]. Datasets with asterisks differ significantly: ^***^p = 0.0007; ^**^p = 0.009.

Significantly fewer FOXO1^+^ signals were identified in CAO compared with CG (*p* < 0.0001, Figure 10A). In addition, the Kruskal–Wallis test showed a significant difference between all three groups (*p* < 0.0001), with the results obtained from early and late arrest samples differing significantly from CG (*p* = 0.0007; late: *p* = 0.009), but no results from early and late arrest differed significantly (Figure 10B).

### 3.4. C-Kit mRNA and protein expression

**Figure 11:**
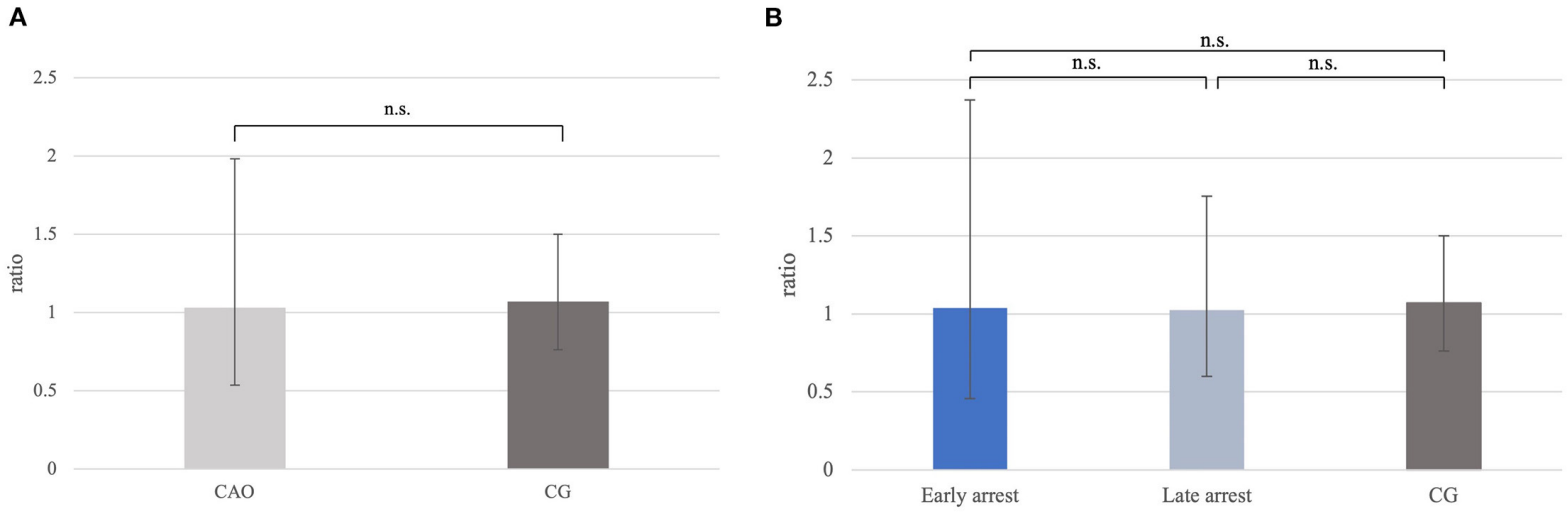
**(A)** *C-Kit* mRNA expression (ratio) in groups of dogs with immune-mediated orchitis (CAO) and healthy control dogs (CG). The results are presented as geometric mean and dispersion factor [$\bar{x}$g (DF)]. **(B)** *C-Kit* mRNA expression (ratio) in dogs with CAO divided into early and late arrest and control dogs (CG). Results are presented as geometric mean and dispersion factor [$\bar{x}$g (DF)].

The ratio for *C-Kit* mRNA expression did not differ significantly between CAO and CG (Figure 11A), nor overall between early and late arrest as well as CG (Figure 11B).

The Western blot for C-Kit revealed a specific immunoreactive band using protein homogenate from an adult dog testis and canine mast cell tumor tissue as positive control; however, the molecular weight of the canine testicular band was slightly higher (approximately 150 kD) than that of the positive control (140 kD) (Figure 2B). Nevertheless, both bands are located within the predicted range of 140-155 kD indicating specificity.

**Figure 12:**
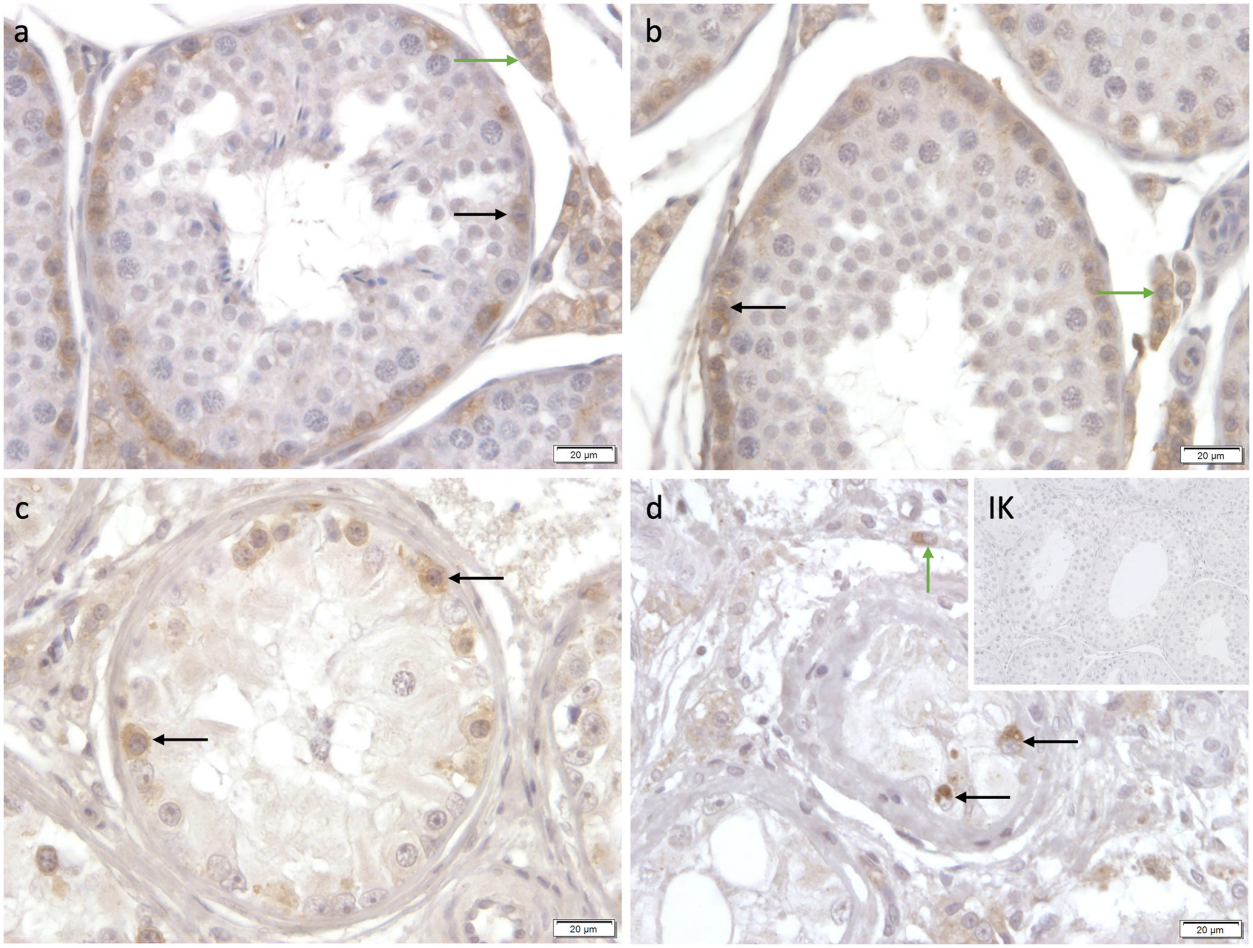
Immunostaining against C-Kit **(a-d)** in CG and CAO dogs (400x magnification). **(a, b)** CG, **(c, d)** CAO [**(c)** early arrest; **(d)** late arrest]. Spermatogonia (*black arrow*); Leydig cells (*green arrow)*.

Specific cytoplasmatic staining against C-Kit was identified in spermatogonia and Leydig cells in CAO and CG. Interestingly, not all spermatogonia stained C-Kit^+^; sporadic cells with prominent round nuclei and extensive cytoplasm, also considered as spermatogonia, did not show any immunopositive signal (Figure 12).

## 4. Discussion

Testicular NOA is the most common cause of infertility in breeding male dogs (5), but also in men (9, 10, 77). Based on our recent findings, idiopathic CAO is an important, but neglected cause of infertility in dogs (8) and men (78). Due to the similarity of findings, such as the lack of clinical signs, lack of the underlying etiology causing immune cell infiltration, and disruption of spermatogenesis, we postulate that CAO in dogs is suitable to gain further insights into human infertility due to CAO. This might be helpful to identify effective treatment options that are still not available for both, dogs (23, 79–84) and men (24, 26, 85–88). Although significantly upregulated PTGS2 expression points to the importance of inflammation as a key process in canine CAO (89), it seems unlikely that selective Cox-2 inhibitors (alone) are suitable to protect fertility indicating the need for other therapeutic options such as assisted reproductive techniques (24, 90, 91). Stem cell-based therapeutic options (36, 42, 92) to (re-) initialize spermatogenesis (37, 38, 40, 47, 93–98) might be a future treatment for CAO-affected dogs, too.

Considering the option of a stem-cell-based therapeutic approach in CAO-affected dogs, knowledge about the presence and possible dysregulation of putative SSCs in canine CAO testes is crucial. Despite the fact that we did not study the final ability of the cells to engraft (47), our results significantly improved the understanding of canine CAO. Confirming the presence of all investigated biomarkers at mRNA and protein levels in CAO-affected canine testes supports our hypothesis that among the detected germ cells resilient SSCs are present—with the potential to replenish the germ cell pool and (re-) initiate spermatogenesis. Further studies should include transplantation assays to finally confirm the true stem cell potential of the surviving cells in canine CAO testes.

In detail, the *PGP9.5* and *DAZL* mRNA ratio of total testicular homogenates and the number of PGP9.5^+^, DAZL^+^, and FOXO1^+^ spermatogonia were significantly decreased in CAO samples compared with normal, healthy testis indicating a severe disruption of spermatogenesis (8). As undifferentiated and differentiating spermatogonia were PGP9.5^+^ in the CAO-affected testis as described in healthy testicular tissues (43, 53, 99), the observed reduction in immunopositive cells supports that all spermatogonial populations are affected by inflammatory and immune-cell mediated changes related to CAO. Similar to previous observations in gracile axonal dystrophic (gad) mice with a deletion within the gene encoding PGP9.5 (100), apoptosis was significantly increased (66) and proliferation of germ cells reduced (unpublished) in canine CAO testis supporting our hypothesis that the accurate regulation of *PGP9.5* mRNA expression is crucial for maintaining canine spermatogenesis, too. Inflammatory changes in canine CAO testis altered PGP9.5 expression; it remains to be clarified whether inflammation is causative for the alteration or a consequence. In addition to the PGP9.5^+^ staining in the early stages of spermatocytes attached to the basal membrane (45), Leydig cells stained positive as previously described for donkeys (99), stallions (101), and men (102), but not for dogs (43). Interestingly, Leydig cells were predominantly stained in CAO samples and especially around the tubules with early arrested spermatogenesis. Surprisingly, another unanticipated PGP9.5^+^ signal was detected in spermatogonia, and preleptotene spermatocytes detached from the basal membrane in the CAO testis. This indicates apoptosis within the early germ cell population, supporting our recent data (66) as well as the postulated PGP9.5 role as a crucial modulator for germ cell apoptosis and sperm quality control during spermatogenesis to maintain testicular homeostasis (99, 103, 104).

The reduced *DAZL* mRNA level in early arrest samples compared with the late arrest further supports that not only the loss (105) but also already a reduction of DAZL might disrupt spermatogonial development and might lead to meiotic arrest at early pachytene. Nevertheless, so far, we cannot determine if the reduced *DAZL* mRNA expression in CAO contributes to the arrest of spermatogenesis or if the reduced number of spermatocytes leads to lower *DAZL* mRNA expression in cases of early arrest, although the latter possibility seems more likely. However, the reduction of *DAZL* mRNA and protein expression during canine CAO has crucial impacts on its role in translational regulation and affects numerous target mRNAs during SSC maintenance and differentiation. Different from the published findings in men and mice (58) indicating a transit of DAZ family protein from the nucleus to cytoplasm during meiosis, we identified DAZL^+^ expression in the cytoplasm of undifferentiated and differentiating spermatogonia and of spermatocytes as observed by Pieri et al. (49) in the dog.

However, *FOXO1* mRNA expression in CAO and CG did not differ, significantly fewer FOXO1^+^ cells were identified in CAO supporting the need for analysis of this target at the protein level (50) due to posttranslational regulation through phosphorylation, acetylation, mono- and polyubiquitination (106). To what extent the posttranslational regulation of FOXO1 is altered during canine CAO has not yet been characterized. FOXO1 protein was localized in undifferentiated spermatogonia in CAO and CG samples reflecting the results of Tarnawa et al. (50) in 11 species studied including the dog. As FOXO1 plays an essential role in SSC maintenance and differentiation in mice, inactivation or loss of FOXO1 causes defects in SSC and therefore spermatogenesis itself (61). Consequently, detecting FOXO1^+^ undifferentiated spermatogonia in the testes of CAO patients has important implications for the prognosis and development of treatment options, as residual FOXO1^+^ cells provide great potential for reinitializing spermatogenesis. Whether the variable staining intensity is biologically relevant and due to defects in SSCs or due to a transition to differentiating spermatogonia, with a decrease and extinction of FOXO1 expression, remains unclear.

Similar to FOXO1, the mRNA level of *C-Kit* did not differ significantly between CAO and CG or between the early or late arrest against CG as shown also in the case of different forms of maturation arrests (107). A possible explanation for this observation could be the relatively high *C-Kit* gene activity in the interstitial cell compartment compared with the intratubular testicular compartment in men (107). As our previous study verified an “enrichment” of the interstitial compartment in CAO testis (8), the missing significance might be attributable to the altered compartment composition supporting the need for laser-assisted cell-picked tissue for mRNA analysis (tubular vs. interstitial compartment). Although C-Kit protein expression subjectively appears to be reduced in the cases of CAO, we did not determine the number of C-Kit^+^ spermatogonia quantitatively. Nevertheless, it seems likely that *C-Kit* mRNA transcription and translation are regulated independently by distant systems, as earlier studies found evidence that undifferentiated spermatogonia can express *C-Kit* mRNA, but not protein (108). C-Kit protein expression and localization seems to differ between species. Whereas, staining of spermatogonia (membranous and cytoplasm), round spermatids (acrosomal), and peritubular Leydig cells was observed in human adult testis (109), germ cells within the stage of type A2 spermatogonia to preleptotene spermatocytes and Leydig cells (110) were C-Kit^+^ in mice and staining in stallion were restricted to differentiating spermatogonia (65). Our results in canine CG and CAO testis with C-Kit^+^ expression in differentiating spermatogonia and Leydig cells were controversial to the observations by Lee et al. (45) detecting C-Kit in spermatocytes differentiating to spermatids and Sertoli cells in adult canine testes, but are in agreement with Grieco et al. (111). However, as the tyrosin-kinase C-Kit and its ligand the stem cell factor (SCF) were postulated to be important modulators of spermatogonial proliferation (112) and differentiation (113) and due to the agreement of observations in human and canine testes, our results confirm C-Kit^+^ expression in a subpopulation of spermatogonia undergoing or committed to differentiation.

Taken together, these findings contribute to our understanding of CAO as accompanied by or possibly even attributable to a significant loss of SSCs. Further research is required to provide deeper insights into the effects of lymphoplasmacytic inflammation and seminiferous tubular degeneration on the maintenance and viability of SSCs. Notwithstanding, our data confirm also for the first time the survival of putative stem cell populations in the damaged testicular tissues probably providing the opportunity for stem cell-based therapeutic options, such as testicular grafting. Further studies are, however, required to investigate this approach in CAO-affected dogs focusing also on possible limitations of testicular grafting (38, 114) in adult dogs with a likely severely disrupted SSC niche due to immune cell infiltration as a cause or consequence of CAO.

## 5. Conclusion

The present study not only enhances our understanding of the pathophysiology of canine CAO but is also the first study revealing the presence of putative SSC markers PGP9.5, DAZL, FOXO1, and C-Kit in CAO-affected canine testicular tissues. Overall, expression of investigated biomarkers was significantly lower in CAO, mostly at the protein level, but also regarding mRNA expressions which supports the hypothesis that immunological and inflammatory changes lead to a disturbance in the maintenance and differentiation of SSCs and consequently to a collapse of testicular homeostasis in CAO-affected dogs. As previously postulated, the CAO tissue is most severely affected in cases with early arrest possibly pointing to the fact that this might be the more “chronic stage” of CAO compared with late arrest. Despite the limitations, these results lay the groundwork for further research into stem cell-based therapeutic options such as testicular xenografting in CAO-affected dogs. Due to the fact that different spermatogonial populations including putative SSCs survive but are unable to provide a continuous output of spermatozoa, further research is required to focus on the surrounding microenvironment, the so-called SSC Niche, to fully understand the implications of chronic CAO on canine spermatogonial stem cells.

## Data availability statement

The raw data supporting the conclusions of this article will be made available by the authors, without undue reservation.

## Ethics statement

Collection of CAO samples for diagnostic purposes was approved by the Dyreforsøgstilsynet Fødevarestyrelsen and by the animal Ethics Committee of the University of Veterinary Medicine Hannover, Foundation. Testis samples from control animals were taken from dogs that were presented for routine castration for other than health issues. Written informed consent was obtained from the owners for the participation of their animals in this study.

## Author contributions

SG-P and LR: conceptualization, sample collection, funding acquisition, and writing–original draft preparation. E-MP, HK, LR, and SG-P: methodology. LR, HK, E-MP, and SG-P: formal analysis, investigation, and writing—reviewing and editing. LR, HK, and SG-P: visualization. SG-P: supervision. All authors contributed to the article and approved the submitted version.
